# Feasibility, Acceptability, and Preliminary Outcomes of a Group‐Based Brief Cognitive‐Behavioral Therapy Protocol for Suicide Prevention in a Brazilian Public Mental Health Service: A Mixed‐Methods Pilot Study

**DOI:** 10.1111/sltb.70143

**Published:** 2026-10-15

**Authors:** Letícia Lopes de Figueiredo, Loren Beiram, Jônatas Magalhães Santos, Mariana Filippini Cacciacarro, Letícia Almeida Silveira Camolesi, Justin C. Baker, Eurípedes Constantino Miguel, Craig J. Bryan, Rodolfo Furlan Damiano

**Affiliations:** ^1^ Instituto de Psiquiatria Hospital das Clínicas da Faculdade de Medicina da Universidade de São Paulo (HCFMUSP) São Paulo SP Brazil; ^2^ Program for Education, Research, and Care in Treatment‐Resistant Depression, Self‐Injury, and Suicidality (Pro‐DRAS) Instituto de Psiquiatria, Hospital das Clínicas da Faculdade de Medicina da Universidade de São Paulo (HCFMUSP) São Paulo SP Brazil; ^3^ Center of Research and Innovation in Mental Health (CISM) São Paulo SP Brazil; ^4^ Department of Psychiatry and Behavioral Health The Ohio State University Columbus Ohio USA; ^5^ Department of Psychiatry University of Vermont Burlington Vermont USA

**Keywords:** Brazil, cognitive‐behavioral therapy, feasibility, group therapy, pilot study, public mental health, suicide prevention

## Abstract

**Introduction:**

We evaluated the feasibility, acceptability, and preliminary outcomes of group‐adapted brief cognitive‐behavioral therapy for suicide prevention (BCBT) in a Brazilian public psychiatric outpatient service.

**Methods:**

This single‐arm, nonrandomized, mixed‐methods pilot enrolled 27 adults across two cohorts receiving 16 weekly group sessions. Feasibility and acceptability were assessed, and suicidal ideation was monitored weekly using the Beck Scale for Suicide Ideation (BSI).

**Results:**

Enrollment targets were met; 70.4% had ≥ 4 recorded sessions, and no intervention‐related adverse events were recorded. All six questionnaire respondents rated recommendation at 8–10. Four provided qualitative responses, highlighting shared suffering recognition and an interpretive theme of hope instillation. The linear BSI slope was nonsignificant (*B* = −0.29, *p* = 0.129); multiple imputation yielded *B* = −0.36 (*p* = 0.018). An exploratory quadratic model indicated greater decline later in treatment (*p* = 0.008). Among 18 participants with ≥ 4 recorded sessions and baseline BSI, scores decreased by 8.2 points from baseline to the last observation (*d* = −0.67; *p* = 0.011).

**Conclusions:**

Group‐adapted BCBT showed preliminary feasibility and acceptability, with uncontrolled signals of reduced suicidal ideation. Controlled evaluation is needed to establish efficacy.

## 1 Introduction

Suicide constitutes a major public health burden, with an estimated 727,000 deaths worldwide in 2021 (World Health Organization 2025). In Brazil, the suicide mortality rate increased by 57% between 2000 and 2019 (Damiano et al. 2024). These trends highlight the need to expand access to evidence‐based, suicide‐specific psychotherapeutic interventions within the Brazilian public mental health system. However, a qualitative study of a Brazilian psychosocial care network identified barriers to psychotherapy access and reported that some group activities functioned primarily as opportunities for prescription renewal (Sampaio and Bispo Júnior 2021).

Brief cognitive‐behavioral therapy for suicide prevention (BCBT), described in foundational work by Rudd (2012) and subsequently manualized by Bryan and Rudd (2018), has accumulated robust evidence of efficacy. In a landmark randomized clinical trial with military personnel, BCBT reduced suicide attempts by 60% over a two‐year follow‐up (Rudd et al. 2015). More recently, a telehealth‐delivered trial demonstrated a 41% reduced risk of attempts compared to present‐centered therapy (Baker et al. 2024), and a subsequent military trial confirmed that BCBT participants were less likely to attempt suicide during follow‐up (Bryan et al. 2025). BCBT has also shown promise in inpatient settings, reducing post‐discharge attempts by 60% (Diefenbach et al. 2024). However, these trials have been conducted almost exclusively in high‐income countries and in an individual‐therapy format.

Critical gaps persist. First, evidence for group‐based delivery of BCBT remains limited. In an early randomized trial of a group‐based problem‐solving intervention among suicidal young adults in a military outpatient setting, both intervention and usual‐care participants improved, while the intervention retained high‐risk participants more effectively (Rudd et al. 1996). More recent work includes a randomized trial protocol comparing group BCBT with dialectical behavior therapy skills groups among military service members (Baker et al. 2023). Second, evidence on psychosocial interventions for self‐harm in LMICs remains limited, particularly regarding culturally adapted interventions and delivery models (Aggarwal et al. 2021). Resource constraints make scalable approaches essential (Patel et al. 2018). Third, routine session‐by‐session monitoring during naturalistic delivery warrants further investigation.

Group delivery may extend access where specialist capacity is limited and provide opportunities for shared learning and peer support (Baker et al. 2023; Yalom and Leszcz 2020). These considerations motivate feasibility testing but do not establish equivalence with individual BCBT. The present pilot evaluated the feasibility, acceptability, and preliminary clinical outcomes of group‐adapted BCBT in a Brazilian public psychiatric outpatient service using mixed methods. It was intended to inform the BCBT‐BR randomized controlled trial, which compares BCBT with supportive psychotherapy at the same site (NCT07574658; Beiram et al. 2027).

## 2 Methods

### 2.1 Study Design and Setting

This single‐arm, nonrandomized, mixed‐methods feasibility pilot, which included exploratory clinical outcomes, evaluated a group BCBT intervention delivered as part of routine clinical care at a university‐affiliated psychiatric outpatient program established in 2024 to integrate specialized care, training, and research on high‐complexity psychiatric conditions, including treatment‐resistant depression, self‐harm, and suicidality. The study involved adults aged 18–65. Group sessions were co‐facilitated by two psychologists trained in cognitive‐behavioral therapy who had completed a BCBT workshop led by a psychologist with expertise in the approach. The therapists participated in weekly supervision with psychiatrists and psychologists. Trainee observers were present during some sessions. After both treatment cohorts had completed the intervention and approval had been obtained from the local ethics committee, outpatient clinic staff who were not involved in data collection explained the study to participants and obtained written informed consent from all participants for the use of their data in this study.

### 2.2 Intervention

The intervention was adapted from the BCBT manual by Bryan and Rudd (2018). The manual and accompanying worksheets and intervention materials were translated into Brazilian Portuguese by one of the authors (LB), without back‐translation. Although individual BCBT and the published group protocol use approximately 12 treatment sessions (Baker et al. 2023; Bryan and Rudd 2018), the local adaptation was planned as a 16‐session group program to allow additional discussion, sharing of individual examples, in‐session practice, review, and consolidation of core BCBT components.

Before the group intervention, each participant completed a separate 90‐min individual session focused on a narrative assessment of their most recent suicidal crisis. This was followed by 16 weekly group sessions lasting approximately 2 h each, including a 10‐min break. The intervention preserved the progression from crisis management and emotion regulation to work on suicide‐related beliefs and relapse prevention. Additional time was devoted to developing reasons for living and the survival kit, practicing cognitive strategies, and reviewing and integrating previously learned skills. Adaptations included a family session addressing psychoeducation, lethal‐means safety, and crisis support; a structured review session using a quiz; and a midpoint feedback session reviewing individual symptom trajectories. The final sessions emphasized skills integration and relapse prevention, culminating in rehearsal of coping responses to a previous suicidal crisis and a hypothetical future crisis.

### 2.3 Participants and Cohorts

Two consecutive cohorts were enrolled. Cohort 1 (*n* = 14) received 16 weekly group sessions from March to July 2024; Cohort 2 (*n* = 13) from September to December 2024. Participants were eligible if they were aged 18–65, were treatment‐seeking outpatients or recently discharged (within 30 days), and reported current suicidal ideation or a history of suicide attempt. Exclusion criteria included acute psychosis or mania, substance intoxication or withdrawal requiring stabilization, severe cognitive impairment, or moderate‐to‐severe intellectual disability. Two participants from Cohort 2 were excluded due to absent BSI data, yielding an analytic sample of *N* = 25. Patients were identified through referrals from services within the hospital complex and from the public healthcare network of the city of São Paulo. A designated outpatient program staff member assessed eligibility for the clinical group using the criteria above.

### 2.4 Feasibility, Acceptability, and Exploratory Clinical Outcomes

Consistent with the conceptual framework for pilot and feasibility studies (Eldridge et al. 2016), the study focused on feasibility and acceptability. Feasibility indicators were recruitment (≥ 10 participants per cohort), retention (≥ 60% completing ≥ 4 of 16 sessions), attendance patterns, and safety (absence of intervention‐related adverse events). A BSI record for a scheduled session was used as a proxy for attendance; missing BSI was treated as absence for these analyses. Retention used all 27 enrolled participants as the denominator, including the two without BSI data.

Acceptability was assessed in Cohort 1 through a post‐intervention questionnaire containing nine five‐point rating items and one recommendation item with response options from 1 to 10 and three open‐ended questions: (1) “How has group therapy impacted your life?”; (2) “Which aspects of the therapy and/or the therapists do you think could be improved?”; and (3) “Is there any other message, suggestion, criticism, or comment you would like to record?” Inter‐member trust, therapists' skills in creating a safe and supportive environment, and overall satisfaction were assessed using separate five‐point items. Recommendation was a separate item; its wording referred to a 0–10 scale, although the available options were 1–10. Other items assessed comfort sharing experiences, cohesion, encouragement of participation, perceived changes in ideation and planning, and usefulness of discussions and activities. Item wording and response anchors are provided in Table S1.

Suicidal ideation was monitored weekly using the Beck Scale for Suicide Ideation (BSI; Beck et al. 1988), completed electronically. The self‐report BSI total comprises items 1–19 (range 0–38); items 20 and 21 concerning past suicide attempts were excluded. BSI trajectories were treated as *exploratory* signal‐detection outcomes to inform the design of a future definitive trial, not as evidence of treatment efficacy.

### 2.5 Data Analysis

Likert‐type items were treated as strictly ordinal (Boone and Boone 2012) and summarized as counts and proportions for the reported response categories. Open‐ended responses were analyzed using content analysis (Bardin 2011) by a single author, Mariana Filippini Cacciacarro (MFC). No second coder was involved; therefore, intercoder agreement was not assessed. For the revised reporting, frequencies were tabulated at the participant level among the four participants who provided open‐ended responses, with each participant counted once per category and categories allowed to overlap. Hope instillation was retained as an interpretive theme from the original content analysis and was not assigned a separate participant frequency. Quantitative and qualitative data were integrated to contextualize findings.

Longitudinal BSI data were analyzed using linear mixed‐effects models with random intercepts and slopes. Session was centered at Session 1. Model 1 estimated linear change; Model 2 added cohort and its interaction with session; Model 3 adjusted linear change for age and sex. Additional exploratory models included quadratic time (Model 4), log (session) with a corresponding random slope (Model 5), and centered baseline BSI and its interaction with session (Model 6). Model 6 included baseline among the repeated outcomes and is descriptive, not an independent test of baseline severity as a predictor. Models 1–5 used 248 observations from 25 participants; Model 6 used 239 observations from 22 participants with baseline BSI. Estimates used restricted maximum likelihood and Satterthwaite degrees of freedom; likelihood ratio tests and AIC/BIC comparisons used maximum likelihood.

Given 38.0% missing BSI values (52.0% at Session 16), sensitivity analysis used multiple imputation by chained equations (MICE; van Buuren and Groothuis‐Oudshoorn 2011): 10 imputations, predictive mean matching, and 20 iterations. Model 7 repeated the linear model in each completed dataset (25 participants, 400 observations) and pooled estimates using Rubin's rules (Rubin 1987). Available‐data models and imputation rely on missing‐at‐random assumptions (Fitzmaurice et al. 2011; Sterne et al. 2009).

Baseline‐to‐last‐observation comparisons used paired *t*‐tests and Wilcoxon signed‐rank tests among participants with ≥ 4 recorded sessions and baseline BSI (*n* = 18). Paired Cohen's d was the mean change divided by its standard deviation. The Reliable Change Index (RCI; Jacobson and Truax 1991) used the scale manual's test–retest reliability of *r* = 0.54 (Beck and Steer 1991). A distribution‐based minimum clinically important difference (MCID; Norman et al. 2003) was estimated as 0.5 × baseline SD = 4.44, rounded up to a five‐point integer threshold. Both classifications used the same 18 participants and the unrounded baseline SD from 22 participants. Analyses used R 4.5.2 (R Core Team 2025), lme4 (Bates et al. 2015), lmerTest (Kuznetsova et al. 2017), and mice.

## 3 Results

### 3.1 Participants and Feasibility Indicators

Table 1 presents baseline characteristics for the 25 participants with BSI data. Mean age was 38.1 years (SD = 11.6), and 72.0% were female. Baseline BSI was 22.8 (SD = 8.9; *n* = 22). Participants had a mean of 9.9 recorded sessions (SD = 5.5). Cohorts did not differ in age (*p* = 0.910), sex (*p* = 1.000), baseline BSI (*p* = 0.498), or attendance (*p* = 0.783). A total of 248 BSI observations were available across 400 possible data points (62.0% completion), with missingness increasing from 12.0% at Session 1 to 52.0% at Session 16 (Figure S1).

**TABLE 1:** Sample characteristics and baseline comparisons.

Characteristic	Overall (*N* = 25)	Cohort 1 (*n* = 14)	Cohort 2 (*n* = 11)	*p*
Age, years, mean (SD)	38.1 (11.6)	38.4 (12.4)	37.8 (11.0)	0.910
Female sex, *n* (%)	18 (72.0)	10 (71.4)	8 (72.7)	1.000
Baseline BSI, mean (SD)	22.8 (8.9)	21.6 (9.6)	24.2 (8.2)	0.498
Recorded sessions attended, mean (SD)	9.9 (5.5)	9.6 (5.6)	10.3 (5.6)	0.783

*Note:* *p* values from Welch's *t*‐tests (continuous variables) and Fisher's exact test (categorical variables). Baseline BSI was available for 22 participants (Cohort 1: *n* = 12; Cohort 2: *n* = 10). Recorded attendance used session BSI completion as a proxy; means describe the 25 participants with BSI data.

Abbreviation: BSI, Beck Scale for Suicide Ideation.

With respect to pre‐defined feasibility criteria, target enrollment was achieved for both cohorts (Cohort 1: *n* = 14; Cohort 2: *n* = 13). Retention was 70.4% (19/27 had ≥ 4 recorded sessions), meeting the pre‐defined threshold of ≥ 60%. Median recorded attendance among these 19 participants was 14 sessions (IQR = 11–15); 18 also had baseline BSI and were eligible for paired analyses. No adverse events attributable to the group intervention were recorded throughout the study period.

### 3.2 Acceptability: Quantitative and Qualitative Findings

The questionnaire was completed by six Cohort 1 participants. All six rated inter‐member trust and therapists' skills in creating a safe and supportive environment at 4–5. Ratings of 4–5 were given by five of six respondents for usefulness of discussions, four of six for decreased ideation, three of six for decreased planning, and five of six for overall satisfaction. All six rated likelihood of recommendation at 8–10.

Four of the six questionnaire respondents answered the open‐ended questions; two left all three questions unanswered. Among these four respondents, two described perceived improvement or a change in perspective, one described acquiring coping tools (“Learning about new tools to cope with difficult days,” P2), and one highlighted recognition of shared suffering (“Seeing that there are people in the same or a worse situation than mine in terms of mental health,” P5). The original content analysis also identified hope instillation as an interpretive theme. All four expressed positive views of the therapists or team. Three suggested changes to treatment delivery: two requested alternative explanations or more time for discussion, and one requested additional attention and continued care. One participant also described discomfort with trainee observers (“I felt a little uncomfortable opening up,” P5). Another indicated that no changes were needed. Table S2 presents participant‐level frequencies and illustrative quotations.

### 3.3 Exploratory Suicidal Ideation Outcomes

Mean BSI decreased from 22.8 (SD = 8.9) at Session 1 to 12.4 (SD = 13.9) at Session 16, with considerable inter‐individual variability (Table 2; Figure 1; Figures S1 and S2). Table 3 summarizes all linear mixed‐effects models for BSI trajectories. The unconditional growth model (Model 1) showed a non‐significant estimated decline in BSI (*B* = −0.29, 95% CI [−0.67, 0.09], *p* = 0.129), with an estimated total change of −4.4 points from Session 1 to Session 16 (95% CI [−10.1, 1.4]). Adding cohort (Model 2) revealed a significant slope for Cohort 1 (*B* = −0.60, 95% CI [−1.09, −0.10], *p* = 0.021) and a non‐significant interaction (*B* = 0.68, 95% CI [−0.06, 1.42], *p* = 0.068), with a significant joint likelihood ratio test for the cohort terms (*χ* ^2^ = 6.25, df = 2, *p* = 0.044). This joint test does not establish a significant session‐by‐cohort interaction. Age and sex (Model 3) were nonsignificant (both *p* > 0.65). The quadratic model (Model 4) suggested greater decline later in treatment (quadratic *B* = −0.04, 95% CI [−0.07, −0.01], *p* = 0.008) and had lower AIC (1570.2 vs. 1575.4) and BIC (1594.8 vs. 1596.5) than Model 1. The log‐time slope (Model 5, *p* = 0.067) and baseline‐by‐session interaction (Model 6, *p* = 0.136) were non‐significant.

**TABLE 2:** BSI descriptive statistics by session.

Session	*N*	Mean (SD)	Median [IQR]	95% CI	Range	% Missing
1	22	22.8 (8.9)	23 [17–31]	[18.8, 26.7]	2–36	12.0
2	19	20.8 (10.2)	24 [12–28]	[15.9, 25.7]	2–35	24.0
3	16	17.9 (12.1)	19 [4–28]	[11.5, 24.4]	2–36	36.0
4	17	21.5 (8.6)	21 [14–30]	[17.1, 26.0]	6–37	32.0
5	19	20.2 (10.4)	19 [14–29]	[15.1, 25.2]	0–37	24.0
6	15	21.2 (9.1)	22 [14–27]	[16.2, 26.2]	5–36	40.0
7	15	15.8 (10.6)	19 [6–24]	[10.0, 21.6]	0–30	40.0
8	14	20.5 (8.2)	21 [18–23]	[15.7, 25.3]	3–36	44.0
9	17	20.1 (11.1)	19 [15–30]	[14.4, 25.8]	1–36	32.0
10	16	20.2 (10.0)	22 [15–27]	[14.9, 25.5]	2–35	36.0
11	13	19.8 (11.0)	22 [13–28]	[13.2, 26.5]	2–33	48.0
12	14	18.8 (11.3)	21 [8–28]	[12.2, 25.3]	0–34	44.0
13	14	20.1 (10.7)	22 [14–30]	[13.9, 26.3]	2–35	44.0
14	14	15.9 (9.6)	18 [8–23]	[10.4, 21.4]	1–32	44.0
15	11	16.8 (12.8)	14 [7–26]	[8.2, 25.4]	1–37	56.0
16	12	12.4 (13.9)	6 [2–18]	[3.6, 21.2]	0–38	52.0

**FIGURE 1:**
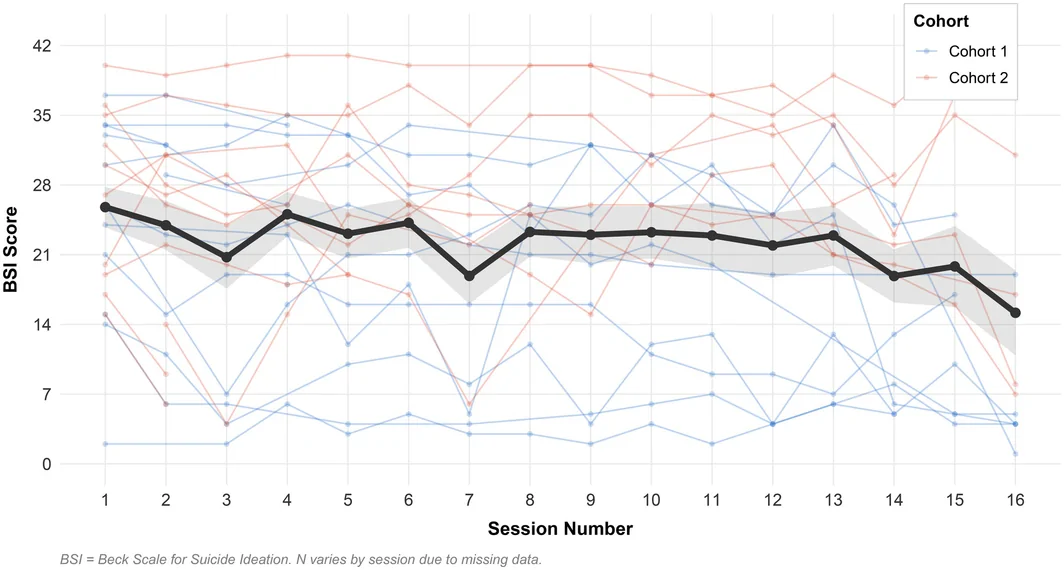
Individual BSI trajectories across group BCBT sessions.

**TABLE 3:** Linear mixed‐effects models for BSI trajectories.

Model	Parameter	B [95% CI]	*p*
Model 1: Linear growth	Intercept	21.22 [16.97, 25.46]	< 0.001
	Session (linear trend)	−0.29 [−0.67, 0.09]	0.129
Model 2: + Cohort interaction	Intercept	20.76 [14.89, 26.63]	< 0.001
	Session (linear trend)	−0.60 [−1.09, −0.10]	0.021
	Cohort 2 (vs. Cohort 1)	1.14 [−7.67, 9.95]	0.792
	Session × Cohort 2	0.68 [−0.06, 1.42]	0.068
Model 3: + Age and sex	Intercept	21.08 [16.06, 26.10]	< 0.001
	Session (linear trend)	−0.28 [−0.67, 0.10]	0.141
	Age (centered)	−0.07 [−0.42, 0.28]	0.670
	Male (vs. Female)	0.47 [−8.60, 9.53]	0.916
Model 4: Quadratic growth	Intercept	20.07 [15.76, 24.38]	< 0.001
	Session (linear trend)	0.29 [−0.26, 0.85]	0.296
	Session^2^ (quadratic)	−0.04 [−0.07, −0.01]	0.008
Model 5: Log‐time growth	Intercept	21.97 [17.46, 26.49]	< 0.001
	log (Session)	−1.58 [−3.28, 0.12]	0.067
Model 6: Baseline severity	Intercept	21.92 [20.30, 23.55]	< 0.001
	Session (linear trend)	−0.36 [−0.71, −0.02]	0.040
	Baseline BSI (centered)	1.10 [0.92, 1.29]	< 0.001
	Session × Baseline BSI	−0.03 [−0.07, 0.01]	0.136
Model 7: MICE‐pooled (*m* = 10)	Intercept	21.58 [18.08, 25.08]	< 0.001
	Session (linear trend)	−0.36 [−0.65, −0.06]	0.018

*Note:* Models 1–6 used REML, random intercepts and slopes, and Satterthwaite df. Models 1–5: 25 participants, 248 observations. Model 6: 22 participants, 239 observations; baseline was also included among the repeated outcomes. Model 7 pooled 10 completed datasets (25 participants, 400 observations each) via Rubin's rules. Likelihood ratio tests and AIC/BIC comparisons used ML. Model 4 had lower AIC and BIC than Model 1.

Abbreviations: B, unstandardized coefficient; BSI, Beck Scale for Suicide Ideation; CI, confidence interval; MICE, multiple imputation by chained equations.

The MICE sensitivity analysis (Model 7) yielded a significant session slope (*B* = −0.36, 95% CI [−0.65, −0.06], *p* = 0.018), corresponding to an estimated change of −5.3 points from Session 1 to Session 16 (95% CI [−9.8, −0.9]; Figure S3). The fraction of missing information was 0.22.

Among 18 participants with at least four recorded sessions and observed baseline and last BSI scores, BSI decreased from 22.7 (SD = 9.0) to 14.5 (SD = 12.8), a mean reduction of 8.2 points (95% CI [2.1, 14.3]; *t* = −2.84, df = 17, *p* = 0.011; Wilcoxon *V* = 33.5, *p* = 0.025; Cohen's d = −0.67). The RCI analysis (threshold: ±16.7 points) classified five participants (27.8%) as reliably improved, 13 (72.2%) as unchanged, and none as deteriorated (Figure 2). Supplementary MCID analysis (threshold: ±5 points) classified nine participants (50.0%) as improved, seven (38.9%) as unchanged, and two (11.1%) as deteriorated (Table S3). The absence of RCI deterioration alone does not establish the safety of the group intervention format.

**FIGURE 2:**
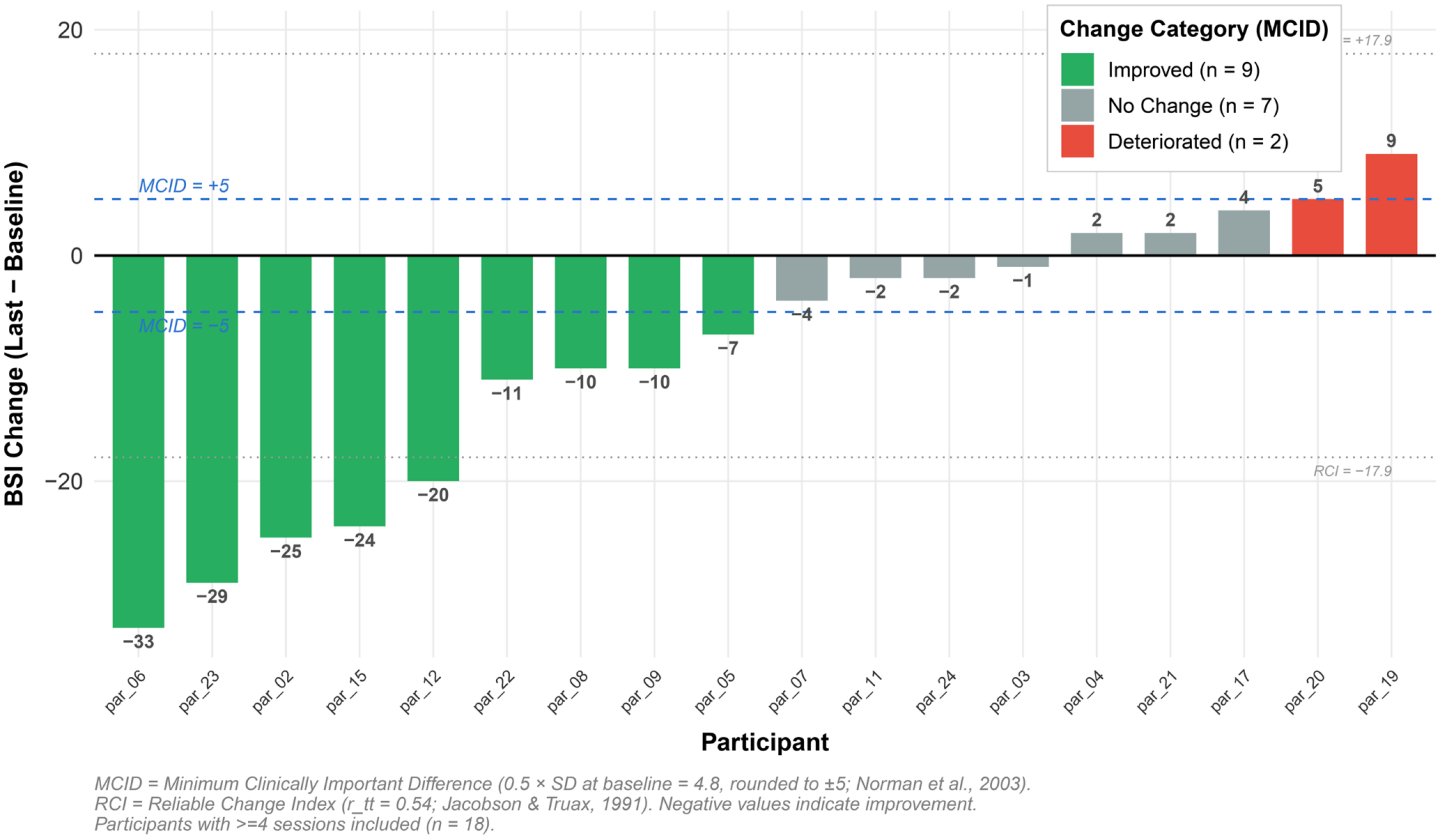
Individual BSI changes from baseline to last assessment.

## 4 Discussion

This pilot provides preliminary evidence on the feasibility and acceptability of group‐adapted BCBT in a Brazilian public psychiatric service. Enrollment targets and the retention threshold were met using the BSI‐based attendance proxy, and no intervention‐related adverse events were recorded. Acceptability ratings among six respondents were favorable, while clinical findings depended on the analytic model. The study supports further controlled evaluation without establishing efficacy or safety.

These acceptability indicators complement the efficacy evidence for individual BCBT (Baker et al. 2024; Rudd et al. 2015). Qualitative findings were interpreted using Yalom and Leszcz's (2020) group therapeutic factors, particularly recognition of shared suffering and hope instillation. Hope instillation reflects the original analyst's interpretation, not a separately quantified or directly measured outcome. One participant's discomfort with trainee observers highlights the need to manage training activities carefully in this setting.

The available‐data linear model estimated a non‐significant decline, whereas multiple imputation and the quadratic model suggested reductions, with the latter indicating greater decline later in treatment. This pattern suggests that a constant linear slope may not fully describe the observed trajectory, but small samples, multiple exploratory models, and missingness limit interpretation. Paired comparisons also suggested improvement, although selection into that subsample restricts inference. The non‐significant cohort interaction does not establish different rates of change between cohorts. None of these analyses establish efficacy or equivalence with individual BCBT.

The uncontrolled design prevents causal attribution; changes may reflect regression to the mean, spontaneous remission, or concurrent treatment. The small sample (*N* = 25) limits precision, and informative missingness remains possible. Attendance was inferred from weekly BSI records, so attendance without form completion or form completion outside a session could be misclassified. The two participants without BSI data remained in the retention denominator as having no recorded sessions. The available data did not support assessment of protocol fidelity, post‐treatment durability, or concomitant‐treatment effects. Translation without back‐translation and the extension to 16 sessions limit assessment of equivalence with the original materials and protocol. Acceptability findings are limited by self‐selection, restriction to Cohort 1, six questionnaire respondents, four open‐ended respondents, and a single qualitative coder without intercoder agreement. Combined questionnaire dimensions, including safety/support and intensity/frequency, cannot be interpreted separately. The distribution‐based MCID is an exploratory benchmark, not a patient‐anchored threshold validated in this sample. Generalizability beyond this university hospital requires further study.

Group‐adapted BCBT warrants further evaluation as a potentially scalable intervention in Brazilian public mental health services. These preliminary feasibility, acceptability, and uncontrolled clinical findings support adequately powered controlled research.

## Author Contributions

**Letícia Lopes de Figueiredo:** conceptualization, investigation, writing – original draft, data curation, formal analysis. **Loren Beiram:** conceptualization, investigation, writing – original draft, visualization, data curation. **Jônatas Magalhães Santos:** writing – review and editing, writing – original draft, formal analysis. **Mariana Filippini Cacciacarro:** conceptualization, investigation, writing – original draft, supervision. **Letícia Almeida Silveira Camolesi:** conceptualization, investigation, writing – original draft, data curation. **Justin C. Baker:** methodology, visualization, validation, writing – review and editing, supervision. **Eurípedes Constantino Miguel:** methodology, validation, visualization, writing – review and editing, supervision. **Craig J. Bryan:** methodology, validation, visualization, writing – review and editing, supervision. **Rodolfo Furlan Damiano:** conceptualization, investigation, writing – original draft, methodology, validation, visualization, writing – review and editing, formal analysis, data curation, supervision.

## Funding

Rodolfo Furlan Damiano received a scholarship from the São Paulo Research Foundation (FAPESP), Brazil (grant #2024/10058‐0).

## Ethics Statement

This study was approved by the local research ethics committee (CAAE: 89774725.0.0000.0068) after completion of both clinical treatment cohorts.

## Consent

All participants provided written informed consent for the use of their clinical data in this research after completion of both treatment cohorts and ethics approval. Outpatient clinic staff who were not involved in data collection explained the study and obtained consent.

## Conflicts of Interest

R.F.D. received honoraria for lectures and educational material from Johnson and Johnson and Viatris.

## Supporting information


**Figure S1:** Session‐by‐session BSI scores and missingness.
**Figure S2:** Mean BSI trajectories by cohort.
**Figure S3:** Sensitivity analysis: Available data vs. multiple imputation.
**Table S1:** Questionnaire items and response options.
**Table S2:** Qualitative findings and illustrative quotations.
**Table S3:** Clinical change classification: RCI and MCID.

## Data Availability

The data that support the findings of this study are available on request from the corresponding author. The data are not publicly available due to privacy or ethical restrictions.
