# Supplementary material for: Feasibility, Acceptability, and Preliminary Outcomes of a Group‐Based Brief Cognitive‐Behavioral Therapy Protocol for Suicide Prevention in a Brazilian Public Mental Health Service: A Mixed‐Methods Pilot Study
Source: Suicide Life Threat Behav. 2026 Oct 15;56(5):e70143. doi: 10.1111/sltb.70143 (PMC13669817; doi:10.1111/sltb.70143)
Supplement: Supplementary file 1 [file SLTB-56-0-s001.docx]

**Supplementary Tables and Figures**

**Table S1. Questionnaire Items and Response Options**

English translations of the questionnaire administered after Cohort 1. Only the endpoints shown below were verbally labeled. Items 1–10 were marked as required in the form; the three open-ended items were optional.

| **Item** | **Question wording** | **Response options and anchors** |
| --- | --- | --- |
| 1 | How would you rate your level of comfort sharing your experiences in the group? | 1–5  1 = very uncomfortable  5 = very comfortable |
| 2 | What level of trust did you feel among group members? | 1–5  1 = little trust  5 = a great deal of trust |
| 3 | How would you rate the overall cohesion of the group? | 1–5  1 = little cohesion  5 = a great deal of cohesion |
| 4 | How would you rate the therapists’ skills in creating a safe and supportive environment? | 1–5  1 = low  5 = high |
| 5 | Did the therapist encourage participation by all group members? | 1–5  1 = not at all  5 = a great deal |
| 6 | Thinking about your suicidal ideation, did you notice that it decreased when comparing the beginning and end of psychotherapy (considering intensity and frequency)? Examples: “I want to go to sleep and never wake up,” “I want to disappear,” “I want to die,” etc. | 1–5  1 = no change  5 = a great deal of change |
| 7 | Thinking about your suicide planning, did you notice that it decreased when comparing the beginning and end of psychotherapy (considering intensity and frequency)? Examples: searching for methods online, developing a concrete plan, etc. | 1–5  1 = no change  5 = a great deal of change |
| 8 | Do you consider that the discussions and activities during the sessions were useful in your process of addressing suicidality? | 1–5  1 = of little use  5 = very useful |
| 9 | What is your overall level of satisfaction with the group therapy experience? | 1–5  1 = very dissatisfied  5 = very satisfied |
| 10 | On a scale of 0 to 10, how much would you recommend this group therapy to a friend or colleague facing similar problems? | Available options: 1–10  No verbal endpoint labels |

Item 10 contains a discrepancy between its wording (0–10) and its available response options (1–10). The manuscript reports the available options. Items 4, 6, and 7 retain their original combined dimensions (safety/support and intensity/frequency); these dimensions were not scored separately.

Open-ended questions: 11. “How has group therapy impacted your life?” 12. “Which aspects of the therapy and/or the therapists do you think could be improved?” 13. “Is there any other message, suggestion, criticism, or comment you would like to record?”

**Table S2. Qualitative Findings and Illustrative Quotations**

Four of the six questionnaire respondents (P2, P4, P5, and P6) answered all three open-ended questions; P1 and P3 left all three unanswered. Frequencies count participants, not mentions, and use a denominator of four. Categories may overlap. Quotations are English translations of the original Portuguese responses.

| **Response category** | **Participants n/N** | **Illustrative quotations** |
| --- | --- | --- |
| Perceived improvement or change in perspective | 2/4  P4, P6 | “I improved considerably, but my case is difficult.” (P4)  “Seeing life differently.” (P6) |
| Acquisition of coping tools | 1/4  P2 | “Learning about new tools to cope with difficult days.” (P2) |
| Recognition of shared suffering | 1/4  P5 | “Seeing that there are people in the same or a worse situation than mine in terms of mental health.” (P5) |
| Alternative explanations or more time for discussion | 2/4  P2, P5 | “I think there should be alternative ways of explaining some topics.” (P2)  “Perhaps a little more time to cover, discuss, and understand the topics more calmly and in greater detail.” (P5) |
| Additional attention and continued care | 1/4  P4 | “I wanted more attention and would like it to continue.” (P4) |
| Discomfort with trainee observers | 1/4  P5 | “As a negative point, in my opinion I do not like having people, trainees, other than the therapists or psychiatrists present in the sessions; I felt a little uncomfortable opening up.” (P5) |
| Positive appraisal of therapists or the treatment team | 4/4  P2, P4, P5, P6 | “I would like to thank the team for their professionalism, innovation, and humanity.” (P2)  “I was with excellent professionals and hope they help more people like me.” (P6) |

Three participants (P2, P4, and P5; 3/4) suggested changes to treatment delivery. P6 stated that no changes were needed. Hope instillation is retained as an interpretive theme from the original content analysis by MFC. The frequencies above describe explicit response content; no separate frequency is assigned to hope instillation.

| **Table S3. Clinical Change Classification: RCI and MCID** | | |
| --- | --- | --- |
| Classification Criterion | n (%) [N = 18] | Method Details |
| **Reliable Change Index (RCI)** |  | **Threshold: ±16.7 points (r_tt = 0.54, SE_diff = 8.51)** |
| Improved | 5 (27.8) |  |
| No Change | 13 (72.2) |  |
| Deteriorated | 0 (0.0) |  |
|  |  |  |
| **MCID (distribution-based, ±5 points)** |  | **Threshold: ±5 points (0.5 × baseline SD = 4.44, rounded up)** |
| Improved | 9 (50.0) |  |
| No Change | 7 (38.9) |  |
| Deteriorated | 2 (11.1) |  |
| *RCI = Reliable Change Index (Jacobson & Truax, 1991); MCID = Minimum Clinically Important Difference (Norman et al., 2003).* | | |
| *RCI based on test–retest reliability r = 0.54 (Beck & Steer, 1991). Both thresholds use the unrounded baseline SD (approximately 8.87; n = 22). MCID = 0.5 × baseline SD = 4.44, rounded up to ±5 points for integer BSI scores; this is a distribution-based benchmark, not a patient-anchored threshold validated in this sample.* | | |
| *Agreement between RCI and MCID classification: 66.7%. Analysis restricted to participants with ≥4 recorded sessions and observed baseline BSI.* | | |

**Figure S1. Session-by-Session** BSI **Scores** and **Missingness**

**Figure S2.** Mean BSI Trajectories by Cohort

**Figure S3.** Sensitivity Analysis: **Available** **Data** vs. Multiple Imputation
